# A survey on the current practice of indicating an elective cesarean after a previous myomectomy

**DOI:** 10.1080/07853890.2023.2197292

**Published:** 2023-04-12

**Authors:** Giovanni Delli Carpini, Valeria Verdecchia, Luca Giannella, Jacopo Di Giuseppe, Barbara Gardella, Pantaleo Greco, Ettore Cicinelli, Andrea Ciavattini

**Affiliations:** a Gynecologic Section, Department of Odontostomatological and Specialized Clinical Sciences, Università Politecnica delle Marche, Ancona, Italy; b Department of Obstetrics and Gynecology, IRCCS Foundation Policlinico San Matteo, Pavia, Italy; c Department of Clinical, Surgical, Diagnostic and Paediatric Sciences, University of Pavia, Pavia, Italy; d Department of Medical Sciences, Obstetric and Gynecological Clinic, University of Ferrara, Ferrara, Italy; e Department of Biomedical and Human Oncological Science, 2nd Unit of Obstetrics and Gynecology, University of Bari, Policlinico, Bari, Italy

**Keywords:** Myomectomy, trial of labor, pregnancy, fibroids, cesarean section

## Abstract

**Objective:**

The objective of this study was to evaluate the attitude of obstetricians/gynecologists toward indicating an elective cesarean delivery in pregnant patients with a previous myomectomy.

**Materials and Methods:**

Web-based multiple-choice questions survey evaluating the attitude to indicate a cesarean with a composite summated score (range 56–280) from a 56-item Likert scale: score 56–112: weak attitude, 113–168: moderate, 169–224: strong, and 225–280: very strong. The reliability of the score (internal consistency) was evaluated with Cronbach’s alpha coefficient. The association between the score and participants’ characteristics was determined with a bivariate analysis followed by linear regression analysis. The “global importance” of each risk factor was defined as the prevalence of the answers: “moderately important”, “very important”, and “extremely important” on the Likert scale. Factors with a “global importance” >75% were considered “crucial” in influencing the choice to indicate a cesarean.

**Results:**

One-hundred-twenty obstetricians/gynecologists responded (response rate 70.6%). The mean ± SD composite summated score was 137 ± 31; 30 (25.0%) participants presented a "weak attitude to cesarean", 68 (56.7%) a "moderate attitude", 22 (18.3%) a "strong attitude", and none a "very strong attitude". The Cronbach’s alpha was 0.934 (high internal consistency). A self-reported number of myomectomies performed per year >50 was associated with a lower score (-25 points, 95% CI −50 to −1, *p* = 0.04). Eight criteria resulted “crucial” in indicating a cesarean: opening of the endometrial cavity, monopolar electrosurgery, time surgery-pregnancy <6 months, 2+ previous myomectomies, hematoma formation in the surgical wound, 3+ removed fibroids, and a FIGO4 or FIGO3 removed fibroid.

**Conclusions:**

Obstetricians/gynecologists are cautious to indicate a cesarean in pregnant patients with a previous myomectomy, except for cases at hypothetic high-risk for uterine rupture, mainly supported by weak evidence. Information to patients and among clinicians is crucial to avoid inappropriate cesarean indications.

**KEY MESSAGES**
- Obstetricians/gynecologists are cautious to indicate a cesarean in pregnant patients with a previous myomectomy.
- Information to patients and among clinicians is crucial to avoid inappropriate cesarean indications in pregnant patients with a previous myomectomy.

## Introduction

Uterine fibroids are the most common benign neoplasm of the female genital tract, and their incidence is directly related to age. Indeed, fibroids are found in 20–25% of women of reproductive age and 30–40% of women older than 40 years [1]. Fibroids could be asymptomatic in most cases; however, they may require a conservative surgical approach (myomectomy) in patients of childbearing age with abnormal vaginal bleeding, pelvic pain, pressure symptoms, or infertility [2]. Furthermore, considering the current trend of delaying childbearing [3,4], the number of pregnant patients with a history of myomectomy will probably rise in the coming years.

Women with a previous myomectomy are at increased risk of uterine rupture during subsequent pregnancies [5]. Although the incidence of uterine rupture after myomectomy is below 1% [6–9], it could significantly affect maternal-fetal mortality and morbidity [6–9]. Therefore, many obstetricians/gynaecologists usually recommend an elective cesarean delivery for pregnant patients with a history of myomectomy to prevent these adverse outcomes. However, very little data exist to support this practice [10]. Moreover, uterine rupture seems to occur more frequently during the third trimester before the onset of labour [11]; therefore, elective cesarean delivery may not prevent these cases.

Excess use of cesarean delivery can lead to harm, as it is associated with an increased risk of short-term and long-term complications [12]. Undoubtedly, there is a need to reduce unnecessary cesarean deliveries, at least through careful evaluation of the indications for elective cesarean deliveries [13]. In this regard, an effective intervention could be to admit women to a trial of labour after myomectomy [14].

However, not all women with a history of myomectomy could benefit from a trial of labour after myomectomy since numerous factors, such as the characteristics of the removed fibroids or the surgical technique, may influence the risk of uterine rupture [6–9,14]. Evidence regarding this risk comes mainly from case reports, case series, or reviews of case reports [6–9,14]. Therefore, it is difficult to make recommendations with adequate quality evidence. This condition could have determined heterogeneous management from the obstetricians/gynaecologists over recent years based on personal experience and using selection criteria supported by weak evidence.

Therefore, this survey aims to evaluate the attitude of obstetricians/gynaecologists toward indicating an elective cesarean delivery in pregnant patients who have had a previous myomectomy, with a specific interest in the factors that may influence the choice of whether or not to perform an elective cesarean.

## Materials and methods

A web-based survey consisting of 69 multiple-choice questions was designed. The questionnaire was addressed to obstetricians/gynaecologists working in Italian hospitals. Questions were clustered into four sections and were constructed to omit phrasing that could influence the answers of participants: (1) Demographics: six questions regarding the participants’ geographical provenience (Northern Italy, Central Italy, or Southern Italy), affiliation to National or International scientific societies, subspecialty (obstetrics, gynaecology, or reproductive medicine), self-reported number of pregnant patients with previous abdominal myomectomy managed per year, self-reported number of myomectomies performed per year, and about previous management of cases of uterine rupture post-myomectomy; (2) Institution: three questions regarding the type of institution (first-level hospital or second-level teaching hospital), the release of documentation about the previously performed myomectomy at the time of hospital discharge, and the possibility that the same operator who performed myomectomy also managed the subsequent pregnancy; (3) General management: four questions on considering a previous myomectomy as an absolute indication of elective cesarean delivery, the trial of labour after myomectomy management, and labour induction; (4) Selection criteria for elective cesarean delivery: participants were asked to rate the importance of the following list of 56 factors (items) that could influence the choice of whether to perform an elective cesarean delivery in pregnant patients who have had a previous myomectomy, using a five-point Likert scale for each factor (1. Not at all important; 2. Slightly important; 3. Moderately important; 4. Very Important; 5. Extremely important):

- Maternal age (<25 years, 26–35 years, 36–45 years, or >45 years)
- Maternal body mass index (BMI) (< 20 kg/m^2^, 20–25 kg/m^2^, 26–30 kg/m^2^, 31–35 kg/m^2^, or >36 kg/m^2^)
- Pregnancy obtained by assisted reproductive techniques (ART)
- Number of removed fibroids (one, two, or three or more removed fibroids)
- Diameter of removed fibroids (< 5 cm, 6–10 cm, 11–20 cm, or > 20 cm)
- Localization of removed fibroids (anterior, posterior, fundal, or lateral)
- Topographic site of removed fibroid according to International Federation of Gynaecology and Obstetrics (FIGO) (FIGO1 to FIGO7)
- Opening of the endometrial cavity
- Surgical technique (laparotomy, laparoscopy, or robotic surgery)
- Type of uterine suture (single layer, double layer, or 3+ layers)
- Type of electrosurgery used (none or minimal use, monopolar, or bipolar)
- Type of surgical thread used for myomectomy (monofilament, twisted, or barbed)
- Duration of myomectomy (<60 minutes, 60–90 minutes, 90–120 minutes, or >120 minutes)
- Hematoma formation in the uterine surgical wound (ultrasound evidence of persistent anechoic and irregular hyperechoic areas in the site of myomectomy) [15,16]
- Number of previous myomectomies (one, two, or more myomectomies)
- Surgical experience of the operator (low, intermediate, or high)
- Time between surgery and pregnancy (< 6 months, 7–12 months, >12 months)
- Maternal will
- Previous vaginal birth.

These criteria were chosen from previous literature [6–9,14] containing reports on the risk factors of uterine rupture in patients who have had a previous myomectomy. Content validity was assessed by a panel of five experts, including only criteria with a content validity ratio (CVR) of 1 [17]. When applicable, all answers contained a “non-response option” to avoid missing data.

The survey was created and administered through Google Forms (Google LLC, Mountain View, California, U.S.A.). Questionnaires were sent to obstetricians/gynaecologists working in Italian hospitals, including structures equally distributed on the national territory and with different complexity of care (first-level hospitals and second-level hospitals), to obtain a representative sample. E-mail addresses were retrieved from public repositories of hospitals. The survey was distributed in May 2021 by an e-mail invitation that contained a brief explanation of the survey, the purpose of the study, the name of the investigator, the duration of the survey, and the policies for data security (see Additional file 1). The e-mail also contained a link to a dedicated form for the processing of personal data and a form for informed consent. The link to the survey was available only after signing the previous forms. A second e-mail invitation was sent to all non-responders after 15 days. All data were collected anonymously, with no monetary incentives. A unique study ID was assigned to each participant to ensure the confidentiality of all self-reported data. Responses were secured using a “Cloud” database (Google LLC, Mountain View, California, U.S.A.) where the data were automatically sorted, scaled, and scored using custom Microsoft Excel formulas (Microsoft Corporation, Redmond, Washington, U.S.A.). Completing the survey was mandatory for all participants who agreed to participate and signed the consent forms. All participants had access to all questions with the same possible responses in the same order. Participants were allowed to answer the questionnaire only once; unique respondents were determined with cookies. Information about cookies was available (Google Forms, Google LLC, Mountain View, California, U.S.A.). The complete survey is available in Additional file 2.

The primary outcome of this survey was represented by a composite summated score that addressed the attitude to indicate an elective cesarean delivery in pregnant women with a previous myomectomy, measured as the sum of the individual responses to each of the 56 items of the Likert scale; the possible score range was 56–280. Four classes of the composite summated score were defined to evaluate the attitude to indicate an elective cesarean delivery: score 56–112 (weak attitude to elective cesarean delivery), score 113–168 (moderate attitude to elective cesarean delivery), score 169–224 (strong attitude to elective cesarean delivery), and score 225–280 (very strong attitude to elective cesarean delivery). The reliability of the summated total score (internal consistency) was evaluated with Cronbach’s alpha coefficient.

The association between the summated total score and the following factors: geographical provenience, type of institution, affiliation to National or International scientific societies, subspecialty, self-reported number of pregnant patients with previous abdominal myomectomy managed per year, self-reported number of myomectomies performed per year, and previous management of cases of uterine rupture post-myomectomy was evaluated using bivariate analysis. Factors that presented an association with the composite summated score with a p-value of <0.05 were considered covariates in a subsequent linear regression analysis.

Results from the Likert scale were also reported as frequencies, separately for each grade of importance (from 1 to 5). We defined the “global importance” of each factor in the choice to indicate an elective cesarean delivery as the sum of the prevalence of the following grades of importance in the answers: “moderately important”, “very important”, and “extremely important”. Factors with a “global importance” greater than 75% were considered “crucial” in the choice to indicate an elective cesarean delivery, while factors with a “global importance” of between 50% and 75% were considered “influential”. Factors with a “global importance” lower than 50% were considered “non-influential”. A diverging stacked bar chart was created for graphical representation and data analysis.

The sample size (n) for the present study was determined with the following equation for determining sample size for Likert scales reported by Park et al. in 2009 [18]:

$$n=\frac{z_{\frac{\alpha }{2}}^{2}\cdot C^{2}}{kD^{2}}\left\{1+\left(k-1\right)\rho \right\}$$

Where k was the number of items of the Likert scale (= 56), C was the coefficient of variation of the population (set at 1), p was the pairwise correlation coefficient (set at 0.5), D was the relative tolerable error (set at 10%), α was set at 0.05, and Z_α/2_ was the 100 (1–α/2)^th^ percentile of the standard normal distribution (= 3.96).

The required sample size (n) derived from the equation was 119 participants. Considering a no-response rate of 30%, the questionnaire was sent to 170 obstetricians/gynaecologists working in hospitals. This study was carried out according to the principles of the Helsinki Declaration of 1975, revised in 2013. The local ethical committee approved the study (Comitato Etico Regionale Marche, CERM, protocol number 2021/34).

Dichotomic variables are reported as numbers and percentages. The normality of each variable was evaluated using the D’Agostino-Pearson test. Normally distributed variables are expressed as arithmetic mean ± standard deviation (SD), while non-normally distributed variables are expressed as the median and interquartile range (IQR). The chi-square test, Mann-Whitney U-test, t-test, or one-way ANOVA was used for variable comparison. The questionnaire responses were downloaded in Microsoft Excel (Microsoft Corporation, Redmond, Washington, U.S.A.) and analyzed using SPSS version 27.0 statistical software (SPSS Inc., Chicago, IL, USA).

## Results

### Demographics

A total of 120 obstetricians/gynaecologists responded to the survey, with a response rate of 70.6%. All participants completed the entire survey. In particular, 28 (23.3%) were from Northern Italy, 73 (60.8%) were from Central Italy, and 19 (15.9%) were from Southern Italy. According to our National organization for obstetrics-gynecologic hospital units, 53 (44.2%) participants worked in first-level hospitals and 67 (55.8%) in second-level teaching hospitals. Sixty-six (55.0%) responders were members of National or International scientific societies. The most frequently reported subspecialty was gynaecology (67, 55.8%), followed by obstetrics (50, 41.7%) and reproductive endocrinology/infertility (3, 2.5%).

The number of self-reported pregnant patients with previous abdominal myomectomy that were managed by each operator per year was between 0 and 10 in 78 participants (65.0%), between 11 and 30 in 35 (29.2%), and between 31 and 50 in seven (5.8%). The participants reported a number of myomectomies performed per year between 0 and 10 in 78 cases (65.0%), between 11 and 25 in 22 (18.3%), between 26 and 50 in 13 (10.8%), and over 50 in seven cases (5.9%). Nine (7.5%) participants reported that they had managed at least one case of uterine rupture post-myomectomy in their careers.

### Institution

Detailed documentation regarding the previously performed myomectomy at the time of hospital discharge was reported to be “always released” from 13 (10.8%) participants, “sometimes released” from 86 (71.7%), and “never released” from the remaining 21 (17.5%).

The participants reported that the same operator who performed the myomectomy also managed the subsequent pregnancy “rarely” in 58.3% of cases, “often” in 40.8%, and “always” in 0.9% of cases.

### General management

A previous myomectomy was not considered an absolute indication of cesarean delivery by 116 (96.7%) of the included obstetricians/gynaecologists, and 96 (80.0%) reported that they had recommended a trial of labour after myomectomy to their patients. Among those, 76/96 (79.2%) indicated that they manage trial of labour after myomectomy according to the trial of labour after cesarean guidelines or protocols, 17/96 (17.7%) used internal protocols of their institution, and 3/96 (3.1%) used their personal experience.

The induction of labour was considered appropriate by 57 (47.5%) operators; 30 (52.6%) of them reported using only mechanical methods for induction of labour, 18 (31.6%), mechanical methods and low doses oxytocin, 2 (3.5%) only low doses oxytocin, 1 (1.8%), prostaglandins only, and 6 (10.5%), mechanical methods, prostaglandins, and low doses oxytocin.

### Attitude to indicate an elective cesarean delivery - composite summated score

The mean ± SD of the composite summated score addressing the attitude to indicate an elective cesarean delivery in pregnant women with a previous myomectomy was 137 ± 31, with a minimum value of 56 and a maximum value of 195. The composite summated score had a high level of internal consistency, as determined by a Cronbach’s alpha of 0.934. According to the four defined classes of attitude, 30 (25.0%) participants presented a “weak attitude to elective cesarean delivery”, 68 (56.7%) a “moderate attitude to elective cesarean delivery”, 22 (18.3%) a “strong attitude to elective cesarean delivery”, and none presented a “very strong attitude to elective cesarean delivery”.

Table 1 reports the factors identified using bivariate analysis to be associated with the composite summated score. The reported subspecialty (gynaecology: 130 ± 31, obstetrics: 143 ± 29, and reproductive endocrinology/infertility: 167 ± 19, *p* = 0.042), the number of self-reported pregnant patients with previous abdominal myomectomy managed per year (<10: 141 ± 29, 11–30: 133 ± 32, 31–50: 106 ± 29, *p* = 0.025), and the self-reported number of myomectomies performed per year (0–10: 143 ± 28, 11-25: 136 ± 36, 25–50: 117 ± 21, >50: 105 ± 33, *p* = 0.002) were associated with the composite summated score with a *p*-value of <0.05 and were included in the linear regression analysis.

**Table 1.** Bivariate analysis of factors associated with the composite summated score for indicating an elective cesarean.

Factor	*n*	Composite summated score	p*
Geographical provenience			
Northern Italy	28	143 ± 29	0.362
Central Italy	73	135 ± 32	
Southern Italy	19	131 ± 27	
Institution			
First-level hospitals	53	139 ± 35	0.365
Second-level teaching hospitals	67	134 ± 28	
Scientific societies			
Member	66	133 ± 29	0.234
Not-member	54	140 ± 33	
Subspecialty			
Gynaecology	67	130 ± 31	0.042
Obstetrics	50	143 ± 29	
Reproductive endocrinology/infertility	3	167 ± 19	
N° of pregnant patients with the previous myomectomy managed per year			
<10	78	141 ± 29	0.025
11–30	35	133 ± 32	
31–50	7	106 ± 29	
N° of myomectomies performed per year			
0–10	78	143 ± 28	0.002
11–25	22	136 ± 36	
25–50	13	117 ± 21	
>50	7	105 ± 33	
Management of at least one case of uterine rupture post-myomectomy			
Yes	9	139 ± 42	0.804
No	111	136 ± 30	

*t-test or one-way ANOVA as appropriate.

The linear regression established that a “self-reported number of myomectomies performed per year > 50” could predict the composite summated score, F (7, 112) = 3.577, *p* = 0.002., adjusted *R*^2^ = 12%. More specifically, a self-reported number of myomectomies performed per year >50 was associated with a lower composite summated score (–25 points, 95% CI −50 points/-1 point, *p* = 0.04).

### Selection criteria for elective cesarean delivery

Table 2 summarizes the importance given by the participants to each selection criteria for elective cesarean delivery. Eight criteria presented a “global importance”>75% and were defined as “crucial” in the choice to indicate an elective cesarean delivery: opening of the endometrial cavity (88.3%), monopolar electrosurgery (88.3%), the time between surgery and pregnancy <6 months (86.7%), two or more previous myomectomies (86.7%), hematoma formation in the uterine surgical wound (81.7%), three or more removed fibroids (79.2%), a FIGO4 removed fibroid (76.7%), and a FIGO3 removed fibroid (75.8%).

**Table 2.** Importance of selection criteria for elective cesarean delivery.

Factor	Global importance (5 + 4 + 3)	Extremely important 5	Very important 4	Moderately important 3	Slightly important 2	Not at all important 1
Maternal age <25 years	4.2%	5 (4.2%)	0 (-)	0 (-)	67 (55.8%)	48 (40.0%)
Maternal age 26–35 years	5.8%	2 (1.7%)	4 (3.3%)	1 (0.8%)	48 (40.0%)	65 (54.2%)
Maternal age 36–45 years	39.2%	4 (3.3%)	27 (22.5%)	16 (13.3%)	25 (20.8%)	48 (40.0%)
Maternal age > 45 years	54.2%	37 (30.8%)	26 (21.7%)	2 (1.7%)	7 (5.8%)	48 (40.0%)
Maternal BMI <20	5.0%	0 (-)	0 (-)	6 (5.0%)	59 (49.2%)	55 (45.8%)
Maternal BMI 20–25	6.7%	0 (-)	0 (-)	8 (6.7%)	55 (45.8%)	57 (47.5%)
Maternal BMI 26–30	10.8%	0 (-)	0 (-)	13 (10.8%)	52 (43.3%)	55 (45.8%)
Maternal BMI 31–35	41.7%	6 (5.0%)	22 (18.3%)	22 (18.3%)	15 (12.5%)	55 (45.8%)
Maternal BMI > 36	45.8%	30 (25.0%)	16 (13.3%)	9 (7.5%)	8 (6.7%)	57 (47.5%)
Pregnancy obtained by ART	37.5%	10 (8.3%)	22 (18.3%)	13 (10.8%)	9 (7.5%)	66 (55.0%
One removed fibroid	6.7%	0 (-)	6 (5.0%)	2 (1.7%)	96 (80.0%)	0 (-)
Two removed fibroids	50.0%	9 (7.5%)	13 (10.8%)	38 (31.7%)	44 (36.7%)	9 (7.5%)
Three or more removed fibroids	79.2%	55 (45.8%)	30 (25.0%)	10 (8.3%)	9 (7.5%)	55 (13.3%)
Diameter of the removed fibroid <5 cm	14.2%	2 (1.7%)	0 (-)	15 (12.5%)	77 (64.2%)	26 (21.7%)
Diameter of the removed fibroid 6–10 cm	61.7%	16 (13.3%)	37 (30.8%)	21 (17.5%)	20 (16.7%)	26 (21.7%)
Diameter of the removed fibroid 11–20 cm	73.3%	46 (38.3%)	31 (23.8%)	11 (9.2%)	6 (5.0%)	26 (21.7%)
Diameter of the removed fibroid >20 cm	73.3%	53 (44.2%)	31 (23.8%)	4 (3.3%)	6 (5.0%)	26 (21.7%)
Anterior localization	30.8%	4 (3.3%)	20 (16.7%)	13 (10.8%)	30 (25.0%)	53 (44.2%)
Posterior localization	15.8%	0 (-)	4 (3.3%)	15 (12.5%)	48 (40.0%)	53 (44.2%)
Fundal localization	33.3%	14 (11.7%)	11 (9.2%)	15 (12.5%)	27 (22.5%)	53 (44.2%)
Lateral localization	21.7%	2 (1.7%)	11 (9.2%)	13 (10.8%)	41 (34.2%)	53 (44.2%)
FIGO1 removed fibroid	35.8%	4 (3.3%)	24 (20.0%)	15 (12.5%)	67 (55.8%)	10 (8.3%)
FIGO2 removed fibroid	69.2%	17 (14.2%)	42 (35.0%)	24 (20.0%)	27 (22.5%)	10 (8.3%)
FIGO3 removed fibroid	75.8%	24 (20.0%)	27 (22.5%)	40 (33.3%)	19 (15.8%)	10 (8.3%)
FIGO4 removed fibroid	76.7%	23 (19.2%)	42 (35.0%)	27 (22.5%)	18 (15.0%)	10 (8.3%)
FIGO5 removed fibroid	34.2%	6 (5.0%)	11 (9.2%)	24 (20.0%)	69 (57.5%)	10 (8.3%)
FIGO6 removed fibroid	16.7%	4 (3.3%)	8 (6.7%)	8 (6.7%)	90 (75%)	10 (8.3%)
FIGO7 removed fibroid	1.7%	2 (1.7%)	0 (-)	0 (-)	108 (90.0%)	10 (8.3%)
Opening of the endometrial cavity	88.3%	62 (51.7%)	28 (23.3%)	16 (13.3%)	2 (1.7%)	12 (10.0%)
Laparotomy	28.3%	8 (6.7%)	7 (5.8%)	19 (15.8%)	47 (39.2%)	39 (32.5%)
Laparoscopy	53.3%	19 (15.8%)	21 (17.5%)	24 (20.0%)	16 (13.3%)	40 (33.3%)
Robotic surgery	38.3%	15 (12.5%)	17 (14.2%)	14 (11.7%)	33 (27.5%)	41 (34.2%)
Single strate	52.5%	23 (19.2%)	25 (20.8%)	15 (12.5%)	14 (11.7%)	43 (35.8%)
Double strate	25.0%	6 (5.0%)	11 (9.2%)	13 (10.8%)	46 (38.3%)	44 (36.7%)
3+ strates	21.7%	9 (7.5%)	9 (7.5%)	8 (6.7%)	57 (47.5%)	37 (30.8%)
None or minimal use of electrosurgery	5.0%	0 (-)	0 (-)	6 (5.0%)	59 (49.2%)	55 (45.8%)
Monopolar	88.3%	62 (51.7%)	28 (23.3%)	16 (13.3%)	2 (1.7%)	12 (10.0%)
Bipolar	30.8%	4 (3.3%)	20 (16.7%)	13 (10.8%)	30 (25.0%)	53 (44.2%)
Monofilament surgical thread	20.0%	4 (3.3%)	17 (14.2%)	3 (2.5%)	13 (10.8%)	83 (69.2%)
Twisted surgical thread	10.8%	5 (4.2%)	6 (5.0%)	2 (1.7%)	23 (19.2%)	84 (70.0%)
Barbed surgical thread	6.7%	2 (1.7%)	3 (2.5%)	3 (2.5%)	25 (20.8%)	87 (72.5%)
Duration <60 min	6.7%	0 (-)	4 (3.3%)	4 (3.3%)	35 (29.2%)	77 (64.2%)
Duration 60–90 min	9.2%	0 (-)	5 (4.2%)	6 (5.0%)	32 (26.7%)	77 (64.2%)
Duration 90–120 min	25.8%	9 (7.5%)	8 (6.7%)	14 (11.7%)	12 (10.0%)	77 (64.2%)
Duration >120 min	29.2%	16 (13.3%)	15 (12.5%)	4 (3.3%)	8 (6.7%)	77 (64.2%)
Surgical hematoma	81.7%	50 (41.7%)	32 (26.7%)	16 (13.3%)	4 (3.3%)	18 (15.0%)
One previous myomectomy	18.3%	2 (1.7%)	4 (3.3%)	16 (13.3%)	89 (74.2%)	9 (7.5%)
Two or more previous myomectomies	86.7%	40 (33.3%)	44 (36.7%)	20 (16.7%)	7 (5.8%)	9 (7.5%)
Low surgical experience	59.2%	35 (29.2%)	22 (18.3%)	14 (11.7%)	0 (-)	49 (40.8%)
Intermediate surgical experience	36.7%	6 (5.0%)	10 (8.3%)	28 (23.3%)	24 (20.0%)	52 (43.3%)
High surgical experience	8.3%	3 (2.5%)	2 (1.7%)	5 (4.2%)	58 (48.3%)	52 (43.3%)
Time surgery – pregnancy <6 months	86.7%	71 (59.2%)	31 (25.8%)	2 (1.7%)	4 (3.3%)	12 (10.0%)
Time surgery – pregnancy 7–12 months	65.8%	10 (8.3%)	28 (23.3%)	41 (34.2%)	29 (24.2%)	12 (10.0%)
Time surgery – pregnancy >12 months	20.8%	2 (1.7%)	9 (7.5%)	14 (11.7%)	83 (69.2%)	12 (10.0%)
Maternal will	55.8%	19 (15.8%)	18 (15.0%)	30 (25.0%)	8 (6.7%)	45 (37.5%)
Previous vaginal birth	37.5%	17 (14.2%)	13 (10.8%)	15 (12.5%)	6 (5.0%)	69 (57.5%)

Data are reported as *n* (%).

**Figure 1.**
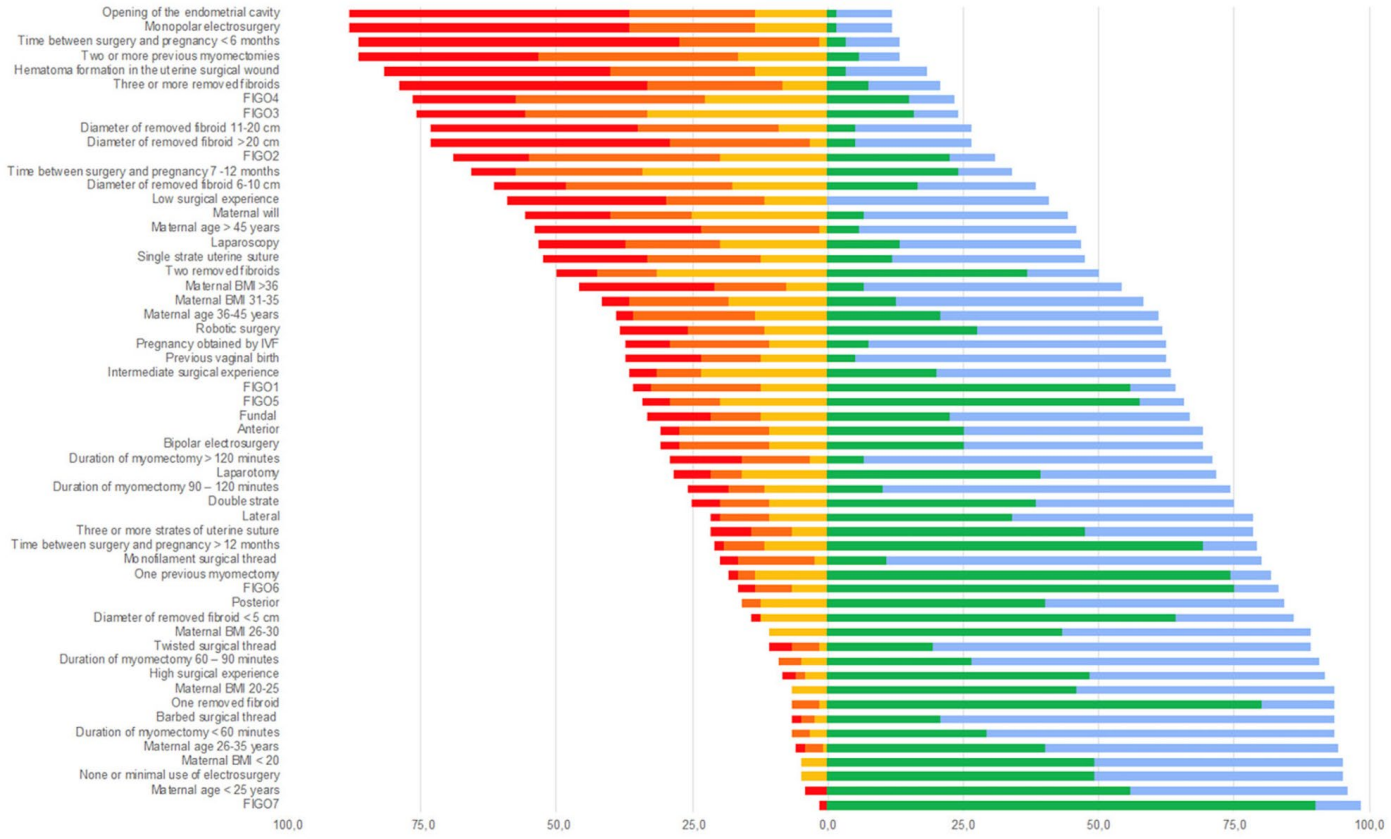
Diverging stacked bar chart of global importance for indicating an elective cesarean delivery after myomectomy.

Figure 1 contains the diverging stacked bar chart about the importance given by the participants to each selection criteria for an elective cesarean delivery.

## Discussion

The present survey showed that most participants (81.7%) were cautious in indicating an elective cesarean delivery in pregnant women who have had a previous myomectomy. This attitude was measured with a composite summated score of 56 items with a Likert scale containing the risk factors for uterine rupture in patients with a previous myomectomy, and it was negatively influenced by the surgical experience of the participant. Indeed, operators who reported that the number of myomectomies performed per year was >50 had lower composite summated scores, indicating a weaker attitude to indicate an elective cesarean delivery in pregnant women with a previous myomectomy. The weaker attitude to indicate an elective cesarean delivery by obstetricians/gynaecologists experienced in the conservative surgical management of uterine fibroids can be explained by the fact that most of the risk factors for uterine rupture are related to surgery. Expert surgeons know in detail the surgical steps and the best techniques that can be used to achieve good healing of uterine sutures (i.e. optimal uterine wall approximation, limited cauterization, less severe tension sutures) [5,6]; therefore, they may be more confident in proposing a trial of labour after myomectomy in patients in whom it is likely to assume that proper uterine healing has occurred. This could be especially true if the same operator who performed the myomectomy also manages the following pregnancies. However, participants reported that this situation occurs “rarely” in many cases (58.3%) and “always” in a small fraction (0.9%). Thus, the patients should always receive a detailed description of surgical procedures with the essential elements useful for future pregnancies. This ensures completeness of information in case of a subsequent pregnancy to provide a correct indication for elective cesarean delivery in cases with crucial risk factors for uterine rupture. Currently, it seems that this practice should be improved, given that the results of this survey show that detailed documentation regarding the previously performed myomectomy was “always released” only in 10.8% of cases, “sometimes released” in 71.7%, and “never released” in 17.5% of cases at the time of hospital discharge.

Among the included selection criteria for elective cesarean delivery, the opening of the endometrial cavity, the use of monopolar electrosurgery, a time between surgery and pregnancy < 6 months, a history of two or more previous myomectomies, hematoma formation in the uterine surgical wound, three or more removed fibroids, and a FIGO3 or FIGO4 removed fibroid were considered as “crucial” in the choice of indicating an elective cesarean delivery, considering that more than 75% of participants rated them as moderately important, very important, or extremely important.

Opening of the endometrial cavity during myomectomy presented a global importance for indicating an elective cesarean delivery of 88.3% (Table 2). This is an expected result, as expert opinions over the past decades supported the choice of an elective cesarean delivery for women in whom the continuity of endometrial cavity was interrupted during a previous myomectomy, assuming that such patients have an increased risk of uterine rupture [5,7,11,19]. However, very little data support this assumption [5,7,11,19,20]. Theoretically, if an opening of the endometrial cavity should remain unrecognized, it could lead to poor uterine healing with a weakened scar and an increased risk of pelvic infection, intrauterine synechiae formation, or adenomyosis [21]. Therefore, the problem could be related to failure to recognize endometrial defects rather than the opening [22], as an accurate suturing technique could prevent those adverse outcomes [19,23–25].

The global importance of monopolar electrosurgery resulted in 88.3%. In comparison, the use of bipolar electrosurgery presented a global importance of 30.8%, and none or minimal use of electrosurgery had a global importance of 5% (Table 2). Excessive use of electrosurgery, particularly monopolar electrosurgery [19,26], is a risk factor for uterine rupture [5–7,21,27–29]. It has also been associated with the weakening of the myometrium, adhesions, poor vascularization, tissue necrosis, thermal damage, tissue hardening, delay in wound healing, increase in collagen deposition, and reduction in smooth muscle fibers, with the formation of an abnormal uterine scar with suboptimal tension, which cannot undergo remodeling during pregnancy, and is less resistant to uterine distension [5–7,10,28]. Thus, several authors indicate that hemostasis during myomectomy should be achieved to avoid excessive electrosurgery, which should be reserved only for single big vessels [6,7,10]. Alternative energy sources are also preferred [5,22], and adjunctive hemostatic techniques (e.g. temporary uterine artery clipping or injection of vasoconstrictors) [30] may be adopted to reduce intraoperative bleeding [6]. However, it is necessary to obtain adequate hemostasis since excessive bleeding in the surgical site may promote hematoma formation and abnormal healing [6,8,21,31].

The time between surgery and pregnancy seems to play a crucial role since if this time is <6 months, 86.7% of participants considered it a moderate, strong, or very strong factor in the choice to indicate an elective cesarean delivery (Table 2). This result may be related to the concern that in cases of a short interval between myomectomy and pregnancy, uterine healing and remodeling processes may not be completed; therefore, the risk of uterine scar rupture may be higher [5]. There is a wide variety of recommendations in the literature regarding the length of the waiting period after myomectomy before attempting to get pregnant, ranging from 3 to 12 months [5,27,32], but these are not supported by valid evidence that addresses the risk of uterine rupture. Imaging studies using ultrasonography, 3D power Doppler ultrasonography, or magnetic resonance [29,32,33] report that the healing process is usually completed within 3–6 months postoperatively. Even if these findings are not correlated with the risk of uterine rupture, they can aid decision-making [5,29]. However, it should be noted that there is probably no safe interval [27,34], and the choice should be individualized according to fertility status and the eventual need for ART [5,29].

In our study, hematoma formation in a uterine surgical wound had a global importance of 81.7%. This condition can occur in cases of inadequate hemostasis or incorrect approximation of the layers of a uterine wound [7,25]. A correct approximation is reported not to be related only to the number of suture layers but to the placement of full-thickness, well-spaced suture, including the deepest layers, avoiding hematoma formation, and without excessive tension on the tissue to prevent ischemia [8,27,29,35]. In addition, hematomas may be associated with abnormal healing and the development of abnormal uterine scarring, along with a higher risk of uterine rupture [7,11,16,28,33,36].

The global importance of a history of two or more previous myomectomies was 86.7%; even if there are no specific data to support this conduct, the recognized rationale is that repeated surgery may further weaken the uterus.

The number and location of fibroids were found to play a crucial role in the choice to indicate an elective cesarean delivery in case of three or more removed fibroids (global importance 79.2%), FIGO3 (global importance 75.8%), or FIGO4 (global importance 76.7%) removed fibroids (Table 2). Some authors have reported those factors as determinants of the quality of the uterine scar [6,9,28]; however, there are insufficient data that can be used to arrive at a solid conclusion [23,37]. Furthermore, more uterine incisions could result in more uterine wall defects, and concerns about FIGO3 or FIGO4 fibroids could be related to the risk of opening the endometrial cavity or the need for extensive repair of defects in the uterine wall.

Weibel et al. surveyed 49 obstetricians in 2014, evaluated their perspectives on labor and delivery after myomectomy, and concluded that breaching the endometrial cavity, regardless of the surgical approach (laparoscopy or laparotomy), was an important factor that influences the choice to perform an elective cesarean delivery, despite the lack of evidence [20]. Our results align with this report since the opening of the endometrial cavity was seen as a crucial factor in our survey, and the surgical approach had a global importance of 53.3% for laparoscopy and 28.3% for laparotomy (Table 2). However, we used more items to evaluate the choice to indicate an elective cesarean delivery, and we included a larger number of participants.

This study had some limitations. One limitation is the potential risk of reduced generalizability of the results of our study, as it involved only obstetricians/gynecologists from a single country, even if they were well distributed throughout the National territory and exhibited varying degrees of competence. Moreover, we cannot completely rule out the risk of selection bias, even if the sample size was determined with a rigorous statistical method [18] and the participants were from structures with homogeneous geographic distribution and a level of complexity of care representative of the national organization.

Furthermore, it is not possible from the data of this survey to draw any indication regarding clinical management, but only conclusions regarding clinicians’ attitudes in daily practice can be made. Currently, no studies can develop an evidence-based approach for managing pregnant women with a previous myomectomy in terms of delivery mode. Indeed, the factors that were found to be “crucial” in indicating an elective cesarean delivery were mainly those not supported by solid evidence. This may be due to the rarity of uterine rupture after myomectomy and the need to include an extremely large number of patients in well-designed clinical studies, a process that seems not feasible from a practical and probably ethical point of view [5]. However, the risk of uterine rupture should not be overlooked, and probably not all patients who have had a previous myomectomy could be eligible for a trial of labor after myomectomy. On the other hand, the risks should not be overemphasized, given that inappropriate indications for cesarean delivery could harm patients. Moreover, most uterine rupture seems to occur before labor, during the third trimester [11,20], and cannot be prevented by an elective cesarean delivery, even if the actual number of uterine ruptures during labor remains unknown, considering the attitude of some obstetricians/gynecologists to propose an elective cesarean delivery, and therefore the inability to collect labor outcomes in these patients.

What seems to emerge from the analysis of the management of pregnant women with a previous myomectomy is that providing information to patients and among clinicians plays a crucial role. First, after a myomectomy, patients who became pregnant should receive adequate counseling regarding the risks associated with their previous surgery from a senior clinician [27]. Second, a detailed transmission of information related to myomectomy from the surgeon to the clinician who will manage the pregnancy is mandatory to collect all available data and appropriately propose the mode of delivery [5]. This is particularly important to avoid indicating a cesarean delivery for patients with a low risk of uterine rupture rather than to indicate with certainty the safety of a trial of labor after myomectomy.

## Conclusions

In conclusion, participants in this survey were cautious about indicating elective cesarean delivery in pregnant women who had a previous myomectomy. The self-reported surgical experience negatively influenced the attitude to indicate an elective cesarean delivery. Most operators recommend performing an elective cesarean delivery in cases in which the endometrial cavity was breached; cases involving the use of monopolar electrosurgery; cases with a short interval between surgery and pregnancy, a history of two or more myomectomies; hematoma formation in the uterine surgical wound; cases of three or more removed fibroids; or cases with FIGO3 or FIGO4 removed fibroids. Therefore, the description of surgical procedures should include the key elements relevant to subsequent pregnancies, and accurate documentation should always be released to the patient.

## Data Availability

The datasets used and analyzed during the current study are available from the corresponding author upon reasonable request.
